# HIV-free children born to HIV-seropositive mothers in Bamako, Mali: a six-year perspective on providing MTCTP at the front line of AIDS

**DOI:** 10.1186/1742-4690-9-S2-P212

**Published:** 2012-09-13

**Authors:** Y Koné, B Aboubacar, L Levitz, C Gomez Mira, J Toffoli, N Ryback, E Kossow, T Huang, A Bicki, K Tounkara, Y Traoré, F Siby Diallo, M Rochas, AS De Groot

**Affiliations:** 1 Community Health Center, ASACOMSI / GAIA Vaccine Foundation, Mékin-Sikoro/ Bamoko, Mali; 2 GAIA Vaccine Foundation, Bamako, Mali; 3 Department of Obstetrics and Gynecology, Hôpital Touré, Bamako, Mali; 4 Regional Department of Health, Bamako, Mali; 5 GAIA Vaccine Foundation, Institute for Immunology and Informatics, Providence, RI, USA

## Background

GAIA Vaccine Foundation (GAIA VF) conducted a six-year retrospective assessment of its Mother To Child Transmission Prevention (MTCTP) program for effectiveness as a non-vaccine HIV prevention tool.

## Methods

The MTCTP program at the Sikoro prenatal care center (Chez Rosalie) opened in 2005. We evaluated MTCTP acceptance and HIV test results of babies born in the MTCTP program from 2005-2011. We also surveyed HIV+ mothers at the clinic about MTCTP risk in July 2011.

## Results

10,471 women were counseled about HIV infection during the study period (average 145/month). An overwhelming majority (99.1%) agreed to HIV testing: 202 (2.15%) were HIV+, of whom 125 (61.9%) accepted MTCTP treatment. Ninety-two HIV+ women delivered at Chez Rosalie. Most used the baby formula provided at the clinic, and a minority chose breastfeeding (as per national policy since 2010). Notably, 100% of babies born to MTCTP-adherent mothers were HIV-seronegative. Thirty-five HIV+ mothers were interviewed about MTCTP for their 150 children. Of the seven polygamous women interviewed, none informed their husbands about their HIV+ status; single and monogamous mothers were significantly more likely to communicate their status.

## Conclusion

The number of women accepting treatment and remaining in care decreased over the 6-year period. Women moved to another clinic after testing positive and also returned to their home villages to deliver, despite having been educated about risks. Two children of mothers who were followed at Chez Rosalie but not enrolled in MTCTP were born HIV+; risk factors for transmitting HIV included late diagnosis (during pregnancy), breast feeding without concomitant ARV treatment, and single parent status. Lack of disclosure was worrisome, considering the number of polygamous relationships. GAIA is working on improving methods to reduce new HIV infections in Sikoro by destigmatizing HIV and MTCTP, and by scaling-up existing peer-education programs to improve willingness to participate in and adhere to MTCTP.

